# Assessment of growth and pain trajectories for children and adolescents receiving chemotherapy for acute lymphoblastic leukemia in Northern Thailand using group-based trajectory modeling

**DOI:** 10.3389/fped.2026.1872012

**Published:** 2026-07-06

**Authors:** Sunisa Phookiaw, Patrinee Traisathit, Sukon Prasitwattanaseree, Chane Choed-Amphai, Lalita Sathitsamitphong

**Affiliations:** 1 Department of Statistics, Faculty of Science, Chiang Mai University, Chiang Mai, Thailand; 2 Data Science Research Center, Department of Statistics, Faculty of Science, Chiang Mai University, Chiang Mai, Thailand; 3 Department of Pediatrics, Faculty of Medicine, Chiang Mai University, Chiang Mai, Thailand

**Keywords:** acute lymphoblastic leukemia, children, group-based trajectory modeling, growth, pain

## Abstract

**Background:**

Children with leukemia often experience growth and symptom changes during treatment. Our aim was to examine bodyweight, body mass index (BMI) and pain trajectories, along with associated factors.

**Methods:**

A retrospective study was conducted among 88 children and adolescents receiving chemotherapy for acute lymphoblastic leukemia (ALL). Group-based Trajectory Modeling was applied to identify trajectory patterns.

**Results:**

Four latent classes for bodyweight trajectory were identified, two-thirds of patients classified into slightly low-normal bodyweight group and mostly were 1–5 years old and in the standard-risk ALL group. Patients in the high-bodyweight group were more likely to be 6–15 years old [adjusted odds ratio (aOR) 184.6 (30.47-1118.48)] and in the high-risk ALL group [aOR 5.36 (1.38–20.8)]. Three latent classes for BMI and pain trajectories were identified. For BMI changes, 94% of patients classified as healthy weight. A minority of patients classified as overweight/obese, which was mainly observed among 6–15-year-old patients [aOR 5.99, (2.18–16.51)] of Thai ethnicity [aOR 4.58 (1.33–15.77)]. Three-quarters of patients experienced high pain initially, and pain levels generally declined over time. High pain was more prevalent in 6–15-year-old patients [aOR 2.61 (1.07–6.36)], whereas mild pain was more common in 1–5-year-old patients and in the standard-risk ALL group. Thai ethnicity was associated with mild pain [aOR 0.36 (0.13–0.99)].

**Conclusions:**

Older age at diagnosis was significantly associated with higher bodyweight, higher BMI and high pain trajectory. The high-risk ALL group was associated with high bodyweight trajectory, and Thai ethnicity was associated with high BMI and mild pain trajectory.

## 1 Introduction

Childhood cancer is a major public health concern in Thailand and worldwide. For children aged 1–14 years old, cancer is the second leading cause of death. The overall incidence of childhood cancer is 140 per million person-years (1), while in Thailand, the incidence of childhood cancer is approximately 98 per million person-years (2). Among childhood cancers, leukemia is the most common, accounting for approximately one-third (28–34.8%) (1–3), of which acute lymphoblastic leukemia (ALL) is the most prevalent (3). Risk-adapted chemotherapy is the mainstay treatment for ALL (4). Although medical advances have significantly improved the treatment success rate, leading to an increase in the 5-year survival rate for childhood ALL in Thailand (5, 6). Children with ALL still encounter significant challenges due to inappropriate growth changes and pain burden resulting from both the disease itself and the treatments or procedures (7–9) and treatment-related complications remain challenging.

A previously published meta-analysis demonstrated unhealthy weight gain and an increase in body mass index (BMI) among pediatric ALL patients during chemotherapy, with many remaining overweight or obese after treatment completion (10). Regarding symptom burden, pain is common during chemotherapy, and uncontrolled pain remains a significant concern (9). Other symptom burdens include fatigue, sleep disturbances, depression, and nausea across all phases of ALL treatment (11). Therefore, monitoring growth changes and pain burden during ALL treatment is crucial, and appropriate management with prompt intervention may lead to better outcomes and improved quality of life.

Cancer-related symptoms during treatment follow a dynamic pattern that can change over time, with symptom severity varying among individual patients (11, 12). To better understand patterns in variability over time, alternative modeling strategies that account for polynomial differences in these changes have been developed. One such approach is Group-based Trajectory Modeling (GBTM), used to identify heterogeneity by classifying individuals with similar trajectories across multiple time points (13–16). Unfortunately, the latest advancements in the application of GBTM have not yet been widely adopted in clinical research (17), and there has been limited research regarding the assessment of growth parameters and pain severity in children with ALL. Exploring these clusters and associated factors during ALL treatment will promote understanding and provide information for proactive strategies to alleviate symptoms. The aim of the present study is to examine growth and pain trajectories, along with their associations with demographic data, focusing on children and adolescents receiving chemotherapy for ALL in Northern Thailand.

## 2 Materials and methods

### 2.1 Study population

Data for the study population were retrospectively reviewed at Chiang Mai University Hospital. These comprised clinical data for children and adolescents diagnosed with ALL at age 1–15 years old and had received chemotherapy across five phases (phase 1: induction, phase 2: consolidation, phase 3: interim maintenance, phase 4: delayed intensification, and phase 5: maintenance) between January 1, 2013, and December 31, 2022. Patients who had not completed all five phases of chemotherapy were excluded from the study.

### 2.2 Data collection and measurements

The demographic data [including age, sex (male/female), and ethnicity (Thai/non-Thai)] and clinical data (including risk stratification [standard risk (SR), high risk (HR) or very high risk (VHR)] based on risk stratification according to the Thai Pediatric Oncology Group (ThaiPOG) Protocol (5) for the study participants were retrospectively collected from their medical records. Bodyweight and height data had been collected across all five phases, while pain scores had been routinely assessed by medical professionals using two distinct age-appropriate pain assessment tools: the Children's Hospital of Eastern Ontario Pain Scale (CHEOPS) (ranging from 4 to 13) and the self-reported Numeric Rating Scale (NRS) (ranging from 0 to 10).

### 2.3 Statistical analysis

This study was divided into two sections based on the data collection approach for children and adolescents with ALL: (1) one-time data collection during phase 1 (induction) (e.g., age, sex, ethnicity, and risk stratification) and (2) repeated measurements across five phases (e.g., bodyweight, height, and pain score). Meanwhile, BMI values were retrospectively calculated based on the bodyweight and height values across all five phases. All parameters were analyzed independently throughout the treatment period.

For the baseline characteristics, categorical variables are presented as the frequency and percentage, while continuous variables are presented as the mean and standard deviation. All participants of non-Thai ethnicity were combined into a single group for analysis to ensure statistical stability and reliable estimates, given the small sample size of each ethnic subgroup. For repeated variables, repeated-measures analysis of variance (RM-ANOVA) was performed as a supplementary descriptive analysis to examine within-subject changes across the five chemotherapy phases and between-group differences across risk stratifications (SR, HR, and VHR). Bodyweight, height, and BMI were standardized to Z-scores that appropriately reflected growth and facilitated the interpretation of growth status in the patients. Because the Children's Hospital of Eastern Ontario Pain Scale (CHEOPS) and the self-reported Numeric Rating Scale (NRS) have different score ranges and measurement properties, direct comparison of raw pain scores across age groups was not appropriate. Therefore, pain scores from both instruments were transformed into standardized T-scores prior to analysis. This transformation was performed by subtracting the mean and dividing by the standard deviation of the respective pain scale, and then linearly rescaling the standardized values to a T-score metric (mean = 50, standard deviation = 10). Standardizing pain scores in this manner placed measurements from both instruments on a common scale with comparable variance, thereby minimizing scale-related bias and enabling the integration of pain data across age groups. This approach enabled pain trajectories to be modeled jointly across chemotherapy phases using Group-based Trajectory Modeling while preserving relative differences in pain intensity within each age group.

GBTM is a semi-parametric technique used to analyze longitudinal data with the aim of classifying individuals into subgroups that exhibit similar trajectories of change in a studied variable over time under the assumption of no within-group variation (18). Individuals are assigned to latent trajectory groups based on observed patterns, such as similar symptoms or behaviors (19–21). GBTM facilitates the clear identification of distinct patterns of change across subgroups and allows for differentiation in the direction of change (e.g., increasing, decreasing, or stable) among groups. This technique can provide a detailed understanding of behavioral or symptomatic changes over time (16, 22, 23). The selection of the number of groups was based on the Bayesian Information Criterion (BIC), clinical interpretability, and the average posterior probability of group membership (recommended > 0.7). For the present study, bodyweight, BMI, and pain had been recorded across five phases of chemotherapy for ALL. GBTM was applied to identify latent classes of symptom trajectories using the pain data and trajectories for physical changes using the bodyweight and BMI data. These continuous variables, which approximately followed a normal distribution, were modeled using the censored normal (CNORM) model. To determine the optimal number of latent classes, both a small change in the BIC and minimal overlap of the 95% confidence intervals (CIs) of the trajectories were considered. After latent classes for bodyweight, BMI, and pain had been identified using GBTM, RM-ANOVA was additionally performed as a supplementary descriptive analysis to examine within-subject changes across the five chemotherapy phases within each identified trajectory group.

We used ordinal logistic regression to determine the relationships between the demographic factors and bodyweight change, BMI, and pain trajectories. This method was applied to estimate the odds ratio representing the probability of greater bodyweight change, BMI change, or more severe pain level. The demographic factors considered in this study included age, sex, ethnicity, and risk stratification. Patient data were separated into two groups for each continuous variable when appropriate. Variables associated with the trajectories with *p* < 0.20 in the univariable analysis were included in the multivariable analysis using the backward elimination method. We checked the proportional odds assumption because ordering in logistic regression is under the assumption that the relationship between the independent variables and all levels of the outcome variable is the same (parallel) across categories. Multicollinearity was evaluated using variance inflation factors (VIFs) based on dummy-coded categorical predictors. We used Stata (version 14) to perform all statistical analyses.

### 2.4 Institutional review board statement

The study was conducted in accordance with the Declaration of Helsinki, and approved by the Research Ethics Committee of Faculty of Medicine, Chiang Mai University (No 052/2024, on FEB 2, 2024) for studies involving humans.

## 3 Results

Of the 88 children and adolescents with ALL, 54 (61.4%) were 1–5 years old, the majority were male (63.6%), and of Thai ethnicity (73.9%). Based on risk stratification, most patients were classified into the SR group (60.2%), followed by the HR group (30.7%) and the VHR group (9.1%). The clinical characteristics of the 88 patients classified based on risk stratification are reported in Table 1.

**Table 1:** Characteristics of the participants who had received chemotherapy for acute lymphoblastic leukemia.

Characteristic	Total (*N* = 88)	Risk Stratification		
		Standard Risk (SR) (*n* = 53)	High Risk (HR) (*n* = 27)	Very High Risk (VHR) (*n* = 8)
**Age (years old)**				
1–5	54 (61.4%)	41 (77.4%)	10 (37.0%)	3 (37.5%)
6–15	34 (38.6%)	12 (22.6%)	17 (63.0%)	5 (62.5%)
**Sex**				
Male	56 (63.6%)	35 (66.0%)	16 (59.3%)	5 (62.5%)
Female	32 (36.4%)	18 (34.0%)	11 (40.7%)	3 (37.5%)
**Ethnicity**				
Thai	65 (73.9%)	45 (84.9%)	16 (59.3%)	4 (50.0%)
Non-Thai	23 (26.1%)	8 (15.1%)	11 (40.7%)	4 (50.0%)

**Figure 1:**
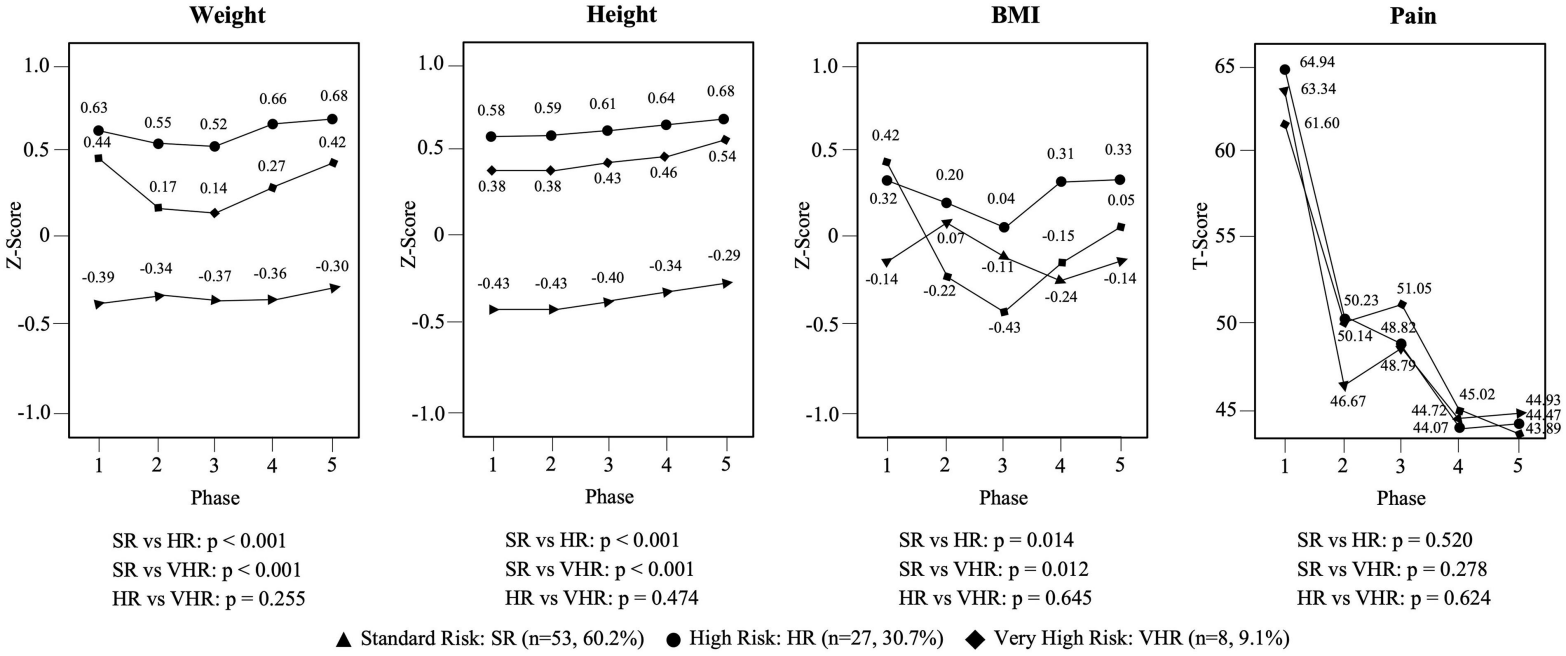
Changes in bodyweight, height, body mass index (BMI), and pain score among children and adolescents who had received chemotherapy for ALL across the risk stratification groups.

Figure 1 presents the overall changes in bodyweight, height, BMI, and pain score among the participants with ALL across the risk stratification groups. In the supplementary descriptive analysis, RM-ANOVA showed a significant main effect of risk stratification on both bodyweight and height (*p* < 0.001). For bodyweight, pairwise comparisons indicate that the patients in the HR and VHR groups experienced weight loss during phase 1 (*p* = 0.015 and *p* < 0.001, respectively), while patients in the SR group underwent weight gain (*p* = 0.036). From phase 3 to phase 5, all three groups experienced weight gain over time. Height increased from phase 1 to phase 5 for all three ALL risk groups. Regarding BMI changes, the SR group showed an increase in BMI during Phase 1 (*p* = 0.001) and a decrease in BMI during Phases 2–4. In contrast, BMI decreased in the HR and VHR groups during Phases 1–3 (*p* = 0.002 and *p* < 0.001, respectively), and then increased afterward in both groups. Comparing bodyweight, height, and BMI changes among the three groups provided significant differences between the SR and HR groups, as well as the SR and VHR groups. However, this was not the case between the HR and VHR groups.

For pain score, although the overall effect of risk stratification was not statistically significant (*p* = 0.615), pairwise comparisons showed a consistent downward trend from phase 1 to phase 2 across all three risk groups (all *p* = 0.001). Notably, in phase 3, patients in the SR and VHR groups exhibited relatively higher pain levels compared to phase 2, albeit not statistically significant (*p* = 0.111 and *p* = 0.790, respectively). Nevertheless, the overall pain score from phase 1 to phase 5 statistically significantly decreased over time (all *p* < 0.001).

### 3.1 Latent classes for the bodyweight trajectory

The optimal number of latent classes for the bodyweight trajectory was determined based on using BIC values. Models with two-to-four latent classes were compared to identify the best-fitting solution. The three-class model showed a lower BIC value (−313.74) compared with the two-class model (−418.56), yielding a *Δ*BIC of 209.64, which indicates the better fit of the former. Subsequently, the four-class model demonstrated an even lower BIC (−282.84) compared with the three-class model (*Δ*BIC = 61.80). Therefore, the four-class model was selected as the optimal solution since it provided the best fit and interpretability for the bodyweight trajectory.

**Figure 2:**
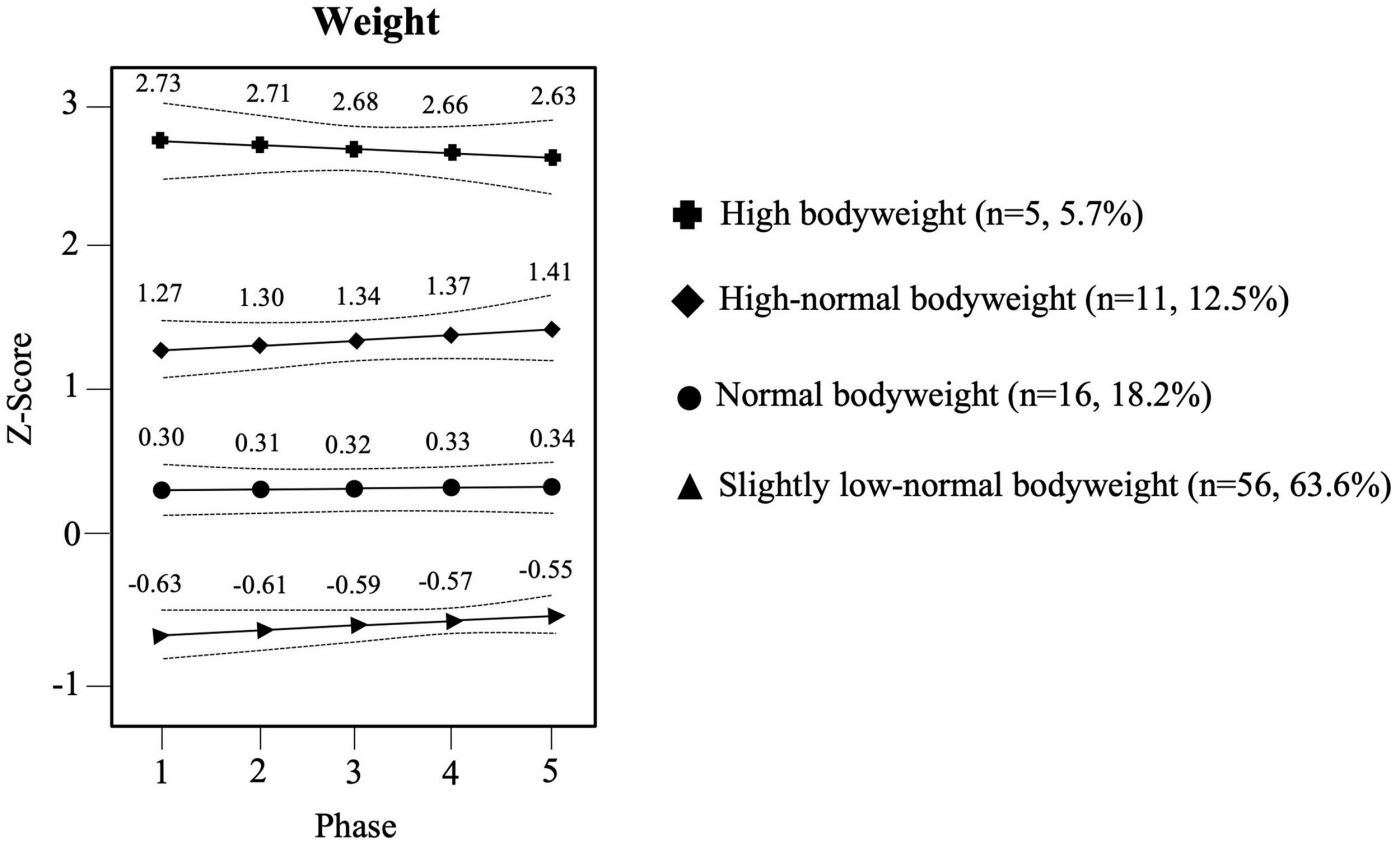
Latent classes for the bodyweight change trajectory in children and adolescents who had received chemotherapy for ALL.

The bodyweight trajectory patterns for the children and adolescents who had received chemotherapy for ALL was stratified into four latent classes, as shown in Figure 2. The terminology and classification of weight Z-scores are shown in Supplementary Table S2. The majority (56 patients; 63.6%) experienced slightly low-normal bodyweight (*p* = 0.230), while 16 (18.2%) had normal bodyweight (*p* = 0.755), 11 (12.5%) had high-normal bodyweight (*p* = 0.307), and 5 (5.7%) had high bodyweight (*p* = 0.626). To further examine within-group temporal changes, pairwise phase comparisons were performed. From phase 1 to phase 5, patients with slightly low-normal bodyweight had gained weight over time (*p* < 0.001). In contrary, patients in the high bodyweight group had weight loss over time (*p* = 0.026). Meanwhile, patients with normal or high-normal bodyweight groups had no statistically significant weight changes across all phases of treatment.

The clinical characteristics of the ALL patients according to the latent classes for the three trajectories are summarized in Supplementary Table S1. Slightly low-to-normal bodyweight was predominantly observed in patients in the SR group and aged 1–5 years old, whereas those with normal or high-normal bodyweight were aged 6–15 years old and distributed across all three ALL risk groups. Meanwhile, those with high bodyweight were all aged 6–15 years and belonged to the HR group.

### 3.2 Latent classes for the BMI trajectory

The optimal number of latent classes for the BMI trajectory was determined based on BIC values. The three-class model showed a lower BIC value (−432.73) compared with the two-class model (−484.72), yielding a *Δ*BIC of 103.98, thereby indicating the better fit of the former model. Unlike the bodyweight trajectory, the four-class model was excluded because one of the trajectory groups contained less than 5% of the total sample, which is considered insufficient for reliable estimation. Therefore, the three-class model was selected as the optimal solution to represent the BMI trajectory.

**Figure 3:**
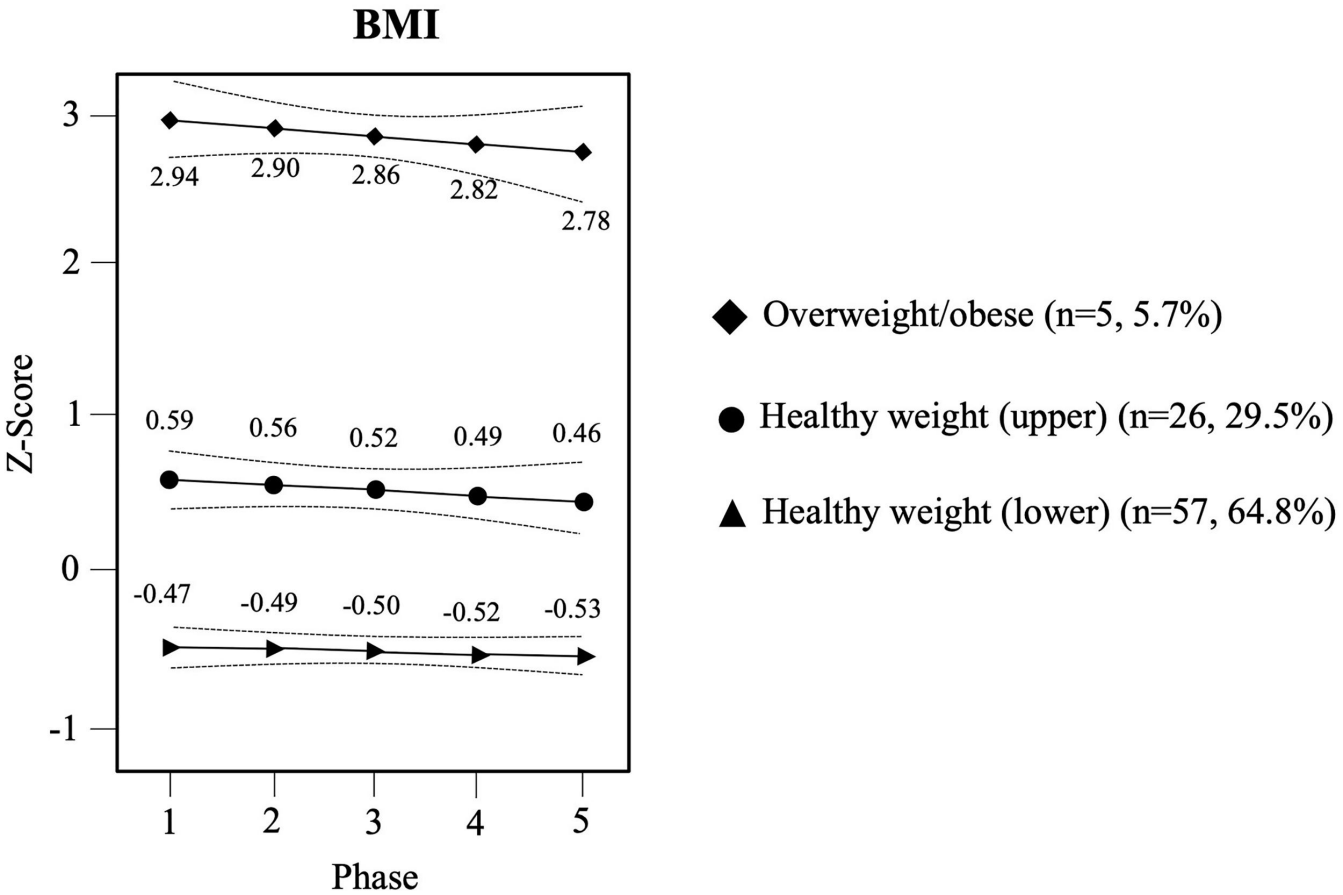
Latent classes for the BMI trajectory in children and adolescents who had received chemotherapy for ALL.

The BMI trajectory patterns for children and adolescents who had received chemotherapy for ALL were separated into three latent classes, as shown in Figure 3. The terminology and classification of BMI Z-scores are shown in Supplementary Table S3. The majority of patients (83; 94.3%) were classified as healthy weight and 5 (5.7%) patients were classified as overweight/obese (*p* = 0.593). Within the healthy weight group, we identified two different latent classes: 57 (64.8%) were in lower healthy weight group (*p* = 0.541), whereas 26 (29.5%) were in higher healthy weight group (*p* = 0.366). To examine within-group temporal changes, pairwise phase comparisons demonstrated a decrease in BMI over time in all three latent classes, albeit not statistically significant. All patients classified as overweight/obese were aged 6–15 years (Supplementary Table S1).

### 3.3 The latent classes for the pain trajectory

The optimal number of latent classes for the pain trajectory was determined based on BIC values. The three-class model showed a lower BIC value (−1478.48) compared with the two-class model (−1483.80), with a *Δ*BIC of 10.64, indicating that the three-class model provided the better fit. Although a four-class model was also examined, it was not selected because one of the trajectory groups contained less than 5% of the total sample, which is considered too small for reliable estimation. Therefore, the three-class model was identified as the optimal solution representing the pain trajectory patterns.

**Figure 4:**
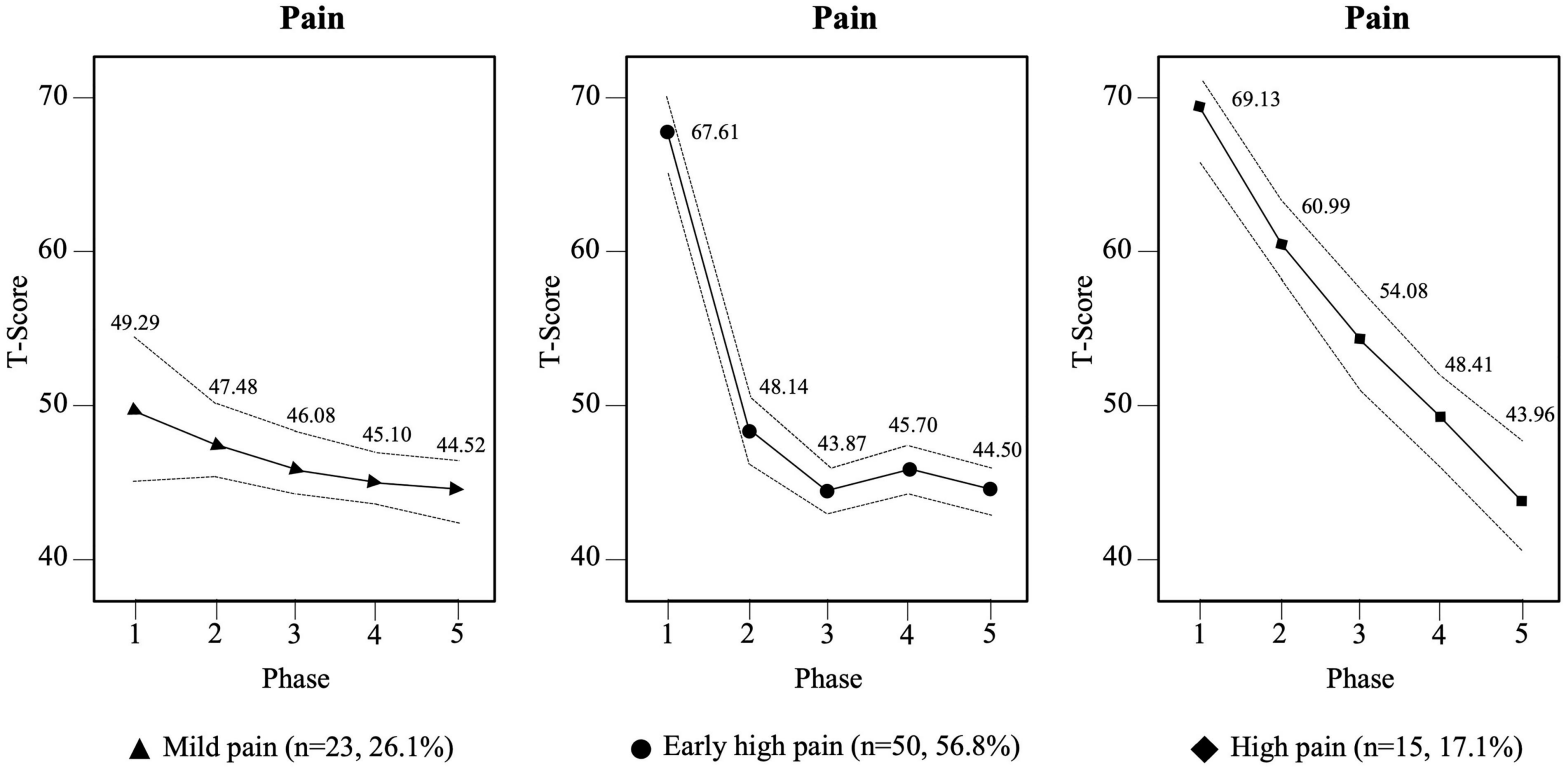
Latent classes for the pain trajectory in children and adolescents who had received chemotherapy for ALL.

Three latent classes for the pain trajectory were applied to the children and adolescents who had received chemotherapy for ALL. The majority of patients (50; 56.8%) experienced early high pain with varying pain levels (*p* = 0.595), 23 (26.1%) experienced mild pain with a decrease in pain level (*p* = 0.267), and 15 (17.1%) patients experienced high pain with a significant decrease in pain level (*p* < 0.001) (Figure 4). To further examine within-group temporal changes, pairwise comparisons between phases showed that pain score significantly decreased over time from phases 1 to 5 according to the three latent classes (all *p* = 0.001). Approximately half of the ALL patients were classified as early high pain, with a significantly high degree of pain reduction from phase 1 to phase 2 in this latent class (*p* < 0.001). Patients with mild pain were predominantly aged 1–5 years old and in the SR group, whereas patients with high pain were mostly male and aged 6–15 years old (Supplementary Table S1).

Table 2 reports the results for the univariable ordinal logistic regression analysis of demographic factors associated with the bodyweight, BMI, and pain trajectories among children and adolescents who had received chemotherapy for ALL. Age and risk stratification were significantly associated with the bodyweight trajectory (*p* ≤ 0.05). Meanwhile, age was a significant risk factor for the BMI trajectory (*p* ≤ 0.05), and although the association of the latter with ethnicity was weaker (*p* < 0.20), it was still within the acceptable limit and so both were included in the multivariable analysis. Similarly, both age and ethnicity were significant for pain trajectory (*p* ≤ 0.05) and were subsequently included in the multivariable model.

**Table 2:** Univariable ordinal logistic regression analysis results for demographic factors associated with bodyweight, body mass index (BMI), and pain trajectories in children and adolescents who had received chemotherapy for acute lymphoblastic leukemia.

Trajectory	Parameter	Univariable Analysis		
		OR	95% CI	*P*
Bodyweight	Age 6–15 vs. 1–5 years old	207.68	35.89–1,201.94	< 0.001
	Female vs. male	0.70	0.29–1.72	0.437
	Thai vs. non-Thai	0.65	0.26–1.66	0.367
	HR vs. SR	7.62	2.79–20.80	< 0.001
	VHR vs. SR	6.20	1.52–25.28	0.011
	VHR vs. HR	0.81	0.20–3.36	0.775
BMI	Age 6–15 vs. 1–5 years old	4.08	1.64–10.19	0.003
	Female vs. male	0.73	0.29–1.81	0.497
	Thai vs. non-Thai	2.53	0.84–7.60	0.098
	HR vs. SR	1.59	0.61–4.11	0.343
	VHR vs. SR	1.18	0.26–5.29	0.830
	VHR vs. HR	0.74	0.15–3.63	0.714
Pain	Age 6–15 vs. 1–5 years old	2.89	1.20–6.99	0.018
	Female vs. male	0.85	0.37–1.98	0.710
	Thai vs. non-Thai	0.32	0.12–0.86	0.024
	HR vs. SR	1.67	0.68–4.13	0.266
	VHR vs. SR	2.58	0.54–12.14	0.231
	VHR vs. HR	1.54	0.31–7.77	0.600

SR, standard risk; HR, high risk; VHR, very high risk.

Table 3 reports the results for the multivariable ordinal logistic regression analysis of demographic factors associated with the bodyweight, BMI, and pain trajectories among children and adolescents who had received chemotherapy for ALL. Given that age is incorporated into the ALL risk stratification system under the ThaiPOG protocols (5) potential multicollinearity between age group and risk category was formally assessed prior to model interpretation: all VIF values were low (range 1.11–1.22; mean VIF = 1.18), verifying the absence of problematic multicollinearity or regression instability in the bodyweight trajectory model.

**Table 3:** Multivariable ordinal logistic regression analysis results for demographic factors associated with bodyweight, body mass index (BMI), and pain trajectories in children and adolescents who had received chemotherapy for acute lymphoblastic leukemia.

Trajectory	Parameter	Multivariable Analysis		
		aOR	95% CI	*P*
Bodyweight	Age 6–15 vs. 1–5 years old	184.60	30.47–1,118.48	< 0.001
	HR vs. SR	5.36	1.38–20.80	0.015
	VHR vs. SR	3.24	0.56–18.86	0.190
	VHR vs. HR	0.61	0.12–3.14	0.550
BMI	Age 6–15 vs. 1–5 years old	5.99	2.18–16.51	0.001
	Thai vs. non-Thai	4.58	1.33–15.77	0.016
Pain	Age 6–15 vs. 1–5 years old	2.61	1.07–6.36	0.035
	Thai vs. non-Thai	0.36	0.13–0.99	0.047

SR, standard risk; HR, high risk; VHR, very high risk.

In the multivariable model for bodyweight trajectory, children and adolescents aged 6–15 years old and in the HR group were significantly more likely to have a higher bodyweight trajectory than those aged 1–5 years old and in the SR group. For the BMI trajectory, age and ethnicity were significantly associated with trajectory group membership in the multivariable analysis (both *p* < 0.05): children and adolescents aged 6–15 years old and of Thai ethnicity were more likely to be in the higher BMI trajectory group compared to those aged 1–5 years old and of non-Thai ethnicity. For the pain trajectory, we found that children and adolescents aged 6–15 years old were significantly more likely to have a higher pain trajectory compared to those aged 1–5 years old, while those with Thai ethnicity were more likely to have a lower pain trajectory than non-Thai patients.

## 4 Discussion

We analyzed changes in bodyweight, height, BMI, and pain scores throughout chemotherapy over five phases in children and adolescents with ALL and categorized them according to risk stratification. This provided detailed insights into differences in trajectory patterns for bodyweight, BMI, and pain scores across various latent classes associated with these trajectories in conjunction with demographic factors.

Regarding bodyweight and BMI trajectories, acute responses to cancer treatment that can influence growth include alterations in energy intake and metabolic balance (24), changes in physical activity, and/or parenting factors (25). Children undergoing cancer treatment may experience treatment-related complications that impair oral intake (e.g., mucositis, nausea, and vomiting), so nutritional support may be required when adequate intake cannot be maintained (26). A dietary evaluation and nutritional intervention should be implemented during cancer treatment to optimize treatment outcomes and minimize complications (27). A study conducted in Mexico demonstrated that one-third of patients with ALL required education and counseling to improve dietary intake, and around 20% required nutritional support to improve weight gain (28). In another study conducted in Croatia (29), around 60% of children with ALL required supplemental nutritional support [either enteral nutrition (EN) or parenteral nutrition (PN)] during treatment; the majority of those experienced weight loss, had significantly lower BMI, and decreased height compared with the non-supplemented control group. Unfortunately, because detailed information regarding dietary intake and nutritional support was not consistently recorded for our retrospective cohort, these variables could not be incorporated into the trajectory models. Therefore, the identified trajectories should be interpreted with caution, as nutritional and other unmeasured clinical factors may have contributed to the observed growth patterns.

Chemotherapy-related side effects (30), as well as medications such as corticosteroids (CS), which are known to increase caloric intake in patients with ALL, have also been associated with subsequent weight gain (25). Arpe ML et al. (31) found that children with ALL had an increased BMI Z-score during the first 150 days of treatment, which was statistically significant when the treatment involved CS. Similarly, Chow EJ et al. (32) reported that higher doses of CS increased BMI and incurred a higher risk of obesity in children with ALL. In a study from Malaysia (33), dietary intake assessment of children with ALL treated via induction and consolidation chemotherapy uncovered that despite consuming less energy, protein, and fat than the control group, the former had higher body fat content than the latter. Medications such as CS play a role in changing body fat composition, with CS administration and dosage influencing weight and BMI changes (Figure 1). In accordance with the ThaiPOG protocol (5), the CS schedule and dosage were similar across all treatment phases for the SR, HR and VHR groups, with a particularly high dosage and a 28-day exposure period during the induction phase. However, patients in the SR group showed weight gain after induction chemotherapy, whereas those in the HR and (most pronouncedly) VHR groups experienced weight loss. This may be due to the greater severity of illness in the latter two groups compared with the SR group during induction, which could have affected weight changes. Additional CS is administered during phases 4 and 5 (during which serious illness is uncommon), which could have influenced the appetites of the ALL patients. Accordingly, after phase 3 (the interim maintenance phase), we observed recovery from weight loss, followed by subsequent weight gain in all ALL-groups. Therefore, monitoring growth parameters, along with ensuring proper nutrition, regular physical activity, and appropriate management of chemotherapy-related complications, can promote good health during treatment.

We found that the height change trend was different compared to those for bodyweight and BMI. Among the groups, we demonstrated a continuous increase in height over time across all chemotherapy phases. On the contrary, Browne et al. (34) reported a continuous reduction in median height z-score during ALL treatment, with recovery of height z-score demonstrated in the off-therapy period. They found that patients aged < 10 years old and those in the low-risk group achieved significant improvement in height z-score after therapy discontinuation. Similarly, Kranjčec I et al. (29) demonstrated a decrease in height Z-score over time during the treatment period, with a predominant decline in height Z-score particularly in high risk ALL, followed by partial recovery after treatment completion. Serious illness, especially cancer and/or inadequate nutrition, can lead to wasting (a short-term response characterized by weight loss). If wasting persists, height stunting may occur. However, the restoration of nutritional status and recovery from serious illness in a timely manner may prevent the development of height stunting (35). These factors may explain the different height changes observed in our cohort. Thereafter, nutritional status evaluation, prompt intervention, and appropriate cancer treatment are principal factors in preventing further wasting and stunting.

In our study, 5.7% of patients were classified as overweight/obese with a decreasing trend. We also demonstrated that patients aged ≥ 6 years old were significantly more likely to have a higher bodyweight and high BMI than those aged 1–5 years old. Similarly, Galati et al. (36) reported a significantly high prevalence of overweight/obese children aged ≥ 10 years old at the time of diagnosis. We demonstrated that patients who were overweight or obese at the time of diagnosis remained on the same trajectory throughout all phases of chemotherapy. This is in agreement with (10, 37), who reported that ALL patients who were overweight/obese at the time of diagnosis remained prone to weight gain over time. Indeed, the prevalence of overweight/obesity appears to increase over time during and after ALL treatment (34). A meta-analysis reported that high BMI or being overweight/obese at the time of diagnosis were associated with obesity in ALL survivors (38). Since children who are overweight/obese are particularly susceptible to cardiovascular-related mortality (39), continual evaluation of bodyweight and BMI during therapy and the off-treatment period is essential, especially in this at-risk group. However, BMI does not necessarily accurately reflecting nutritional status. To overcome this limitation, basic and advanced body composition assessments are useful for evaluating muscle and adipose tissue, thereby providing more specific measures for identifying malnutrition, overweightness/obesity, sarcopenia, and sarcopenic obesity (40, 41). This highlights the importance of monitoring growth and body composition, encouraging physical activity, and providing appropriate nutritional guidance during treatment. Moreover, health education should be promoted, and appropriate interventions should be implemented promptly when indicated, both during treatment and afterward.

Although pain is a common problem during cancer treatment, cancer-related pain is uncommon in childhood cancers (42, 43). We demonstrated that pain scores were highest at treatment initiation and declined across all groups, particularly between phases 1 and 2 after chemotherapy initiation. In newly diagnosed ALL, both chemotherapy treatment and painful procedures, such as bone marrow aspiration and lumbar punctures, have been reported to influence pain intensity (42–44). Similarly, studies conducted in the USA and Germany have demonstrated a continuous decline in pain levels over time during treatment (45). Thus, pain should be effectively managed as treatment progresses, even though fluctuations in pain intensity may occur due to variations in treatment intensity and chemotherapy-related side effects, especially mucositis (30). Regarding pain trajectory, approximately half of the patients experienced early high pain with fluctuating levels, while the others showed a steady decrease in pain score throughout all treatment phases. Similarly, Hockenberry et al. (11) also reported that half of the cohort experienced moderate symptoms that decreased over time, while 11.1% experienced severe symptoms that also declined significantly. Interestingly, we found that patients in all three latent pain-trajectory classes exhibited a decline in pain score over time, which may reflect physiological adaptation, improved symptom management, and/or psychological adjustment to pain stimuli (46).

In our study, ALL patients aged 6–15 years had significantly higher pain levels than those who were younger. As previously reported (47), pain is an essential symptom burden during cancer treatment, particularly in emotionally distressed adolescents. A prospective study of parents' perception of adolescent found that pain in adolescents was greater than that in young children (45). In the latter group, pain evaluation is quite difficult, and under-expression of pain may explain the low frequency of pain burden in this group. Furthermore, mild pain was more common among patients aged 1–5 years and those in the SR group. This finding may be related to the side effects of chemotherapy and painful procedures during treatment (42–44). Variations in chemotherapy intensity among the different groups of ALL classification influence the treatment-related adverse events. Patients in the HR and VHR groups experienced higher pain due to more intensive chemotherapy, higher treatment-related pain, and more frequent painful procedures compared to those in the SR group. This finding highlights the importance of pain management strategies, especially during induction chemotherapy, when patients undergo various painful procedures. Moreover, pain resulting from mucositis is another concern (30), as it may lead to limited oral intake and subsequent weight loss. Therefore, strategies for pain monitoring and appropriate pain management are essential components of clinical practice.

Moreover, we demonstrated that ethnicity was a significant factor associated with changes in BMI and pain severity. Socioeconomic status and environmental factors may influence growth, health and well-being among different ethnic groups. This was highlighted by Hockenberry et al. (11), who reported that the experience of pain severity was significantly different between Hispanic and non-Hispanic patients. Moreover, previous studies have suggested that cultural beliefs, communication styles, pain-coping behaviors, family support, socioeconomic circumstances, biological factors, and access to supportive care resources may contribute to differences in pain perception and pain management outcomes across ethnic groups (48–50). However, the present study was not specifically designed to investigate the mechanisms underlying the effect of ethnicity on pain perception, and so this finding should be interpreted cautiously. Further prospective studies are needed to better understand these complex interactions in the pediatric oncology setting.

The present study highlights distinct trajectories in bodyweight, BMI, and pain among children and adolescents who had undergone chemotherapy for ALL. These patterns can be effectively used to assess growth changes and pain severity, thereby aiding healthcare planning and growth monitoring during treatment to prevent complications and enhance quality of life. However, our study has several limitations. First, applying GBTM is a challenge, particularly in determining the optimal number of groups. This requires a balance between statistical criteria (e.g., BIC) and clinical judgment to ensure both statistical precision and clinical relevance (17, 51). Another concern is the imbalance in sample sizes across the latent classes, which could have affected the stability and precision for parameter estimation and reduced the statistical power to detect between-group differences. In particular, one trajectory group contained only a small number of participants (*n* = 5), which may have contributed to unstable regression estimates in the subsequent ordinal logistic models. It should also be noted that, while distinct classes for the pain trajectory were identified, the groups exhibited relatively similar characteristics for the later treatment phases (particularly phases 4 and 5). This overlap could have affected the group classification accuracy and the reliability of the estimated associations in the ordinal logistic regression. Future studies with larger sample sizes and/or alternative modeling techniques could help clarify these group differences.

Apart from restricting the statistical analysis, the retrospective study design was affected by incomplete data and limited available clinical information. The lack of nutritional information concerning dietary intake, diet diaries, and enteral or parenteral nutritional support, which can influence weight and BMI changes during ALL treatment, led to insufficient evidence to establish conclusive associations with growth changes. Furthermore, body composition assessment, which provides a more specific evaluation of nutritional status, was not routinely performed. Therefore, future studies incorporating body composition analysis are warranted, and the identified growth trajectories should be interpreted with caution. In our cohort, only pain score could be analyzed. Exploring complete symptom burden during ALL treatment is crucial. Therefore, future research should incorporate a more comprehensive symptom analysis, including factors such as fatigue, sleep disturbance, and nausea and vomiting, thereby enabling the application of Multitrajectory Modeling. This approach enables the simultaneous analysis of multiple symptoms over time, thereby providing a more holistic understanding of the disease course and treatment impact. Finally, the relatively small sample size may have limited the statistical power, reduced the precision of the subgroup analyses, and restricted comparisons of trajectory patterns across the ALL risk stratification groups (SR, HR, and VHR). Because these groups differ in treatment intensity and treatment-related side effects, further studies with a larger sample sizes are needed to better characterize potential differences in growth and symptom trajectories when applying risk stratification. Such studies may enhance our understanding of these patterns and contribute to improved treatment planning and supportive care.

## 5 Conclusions

Group-based Trajectory Modeling has been applied to analyze and characterize heterogeneity in growth patterns and pain trajectories in research settings, as well as to identify at-risk groups and associated factors. In our pediatric ALL cohort, older age at diagnosis and Thai ethnicity were significantly associated with being overweight or obese, and the HR group was associated with higher bodyweight compared with the SR group. We demonstrated that patients who were overweight or obese tended to remain in the same weight category over time. This group requires appropriate intervention and monitoring. In addition, older age at diagnosis was associated with higher pain severity, whereas Thai ethnicity was associated with lower pain severity. These findings highlight the importance of effective pain management strategies and policies throughout treatment. To better understand these trajectories among children and adolescents being treated for ALL, a prospective comprehensive study is required to elucidate differences in these trends and to identify the associated factors, including demographic characteristics, treatment-related factors and outcomes, dietary intake, nutritional support, and physical activity during treatment, for improving patient monitoring and ensuring timely and appropriate interventions for individuals at risk.

## Data Availability

The data analyzed in this study is subject to the following licenses/restrictions: The data presented in this study are available on request from the corresponding author. Requests to access these datasets should be directed to Lalita Sathitsamitphong, MD, lalita.sat@cmu.ac.th.
