# Supplementary material for: Assessment of growth and pain trajectories for children and adolescents receiving chemotherapy for acute lymphoblastic leukemia in Northern Thailand using group-based trajectory modeling
Source: Front Pediatr. 2026 Jul 6;14:1872012. doi: 10.3389/fped.2026.1872012 (PMC13381744; doi:10.3389/fped.2026.1872012)
Supplement: Supplementary file 1 [file Table1.docx]

Supplementary Material

# Supplementary Tables

**Supplementary Table 1.** The numbers and percentages of children and adolescents who had received chemotherapy for ALL according to the bodyweight, BMI, and pain trajectory groups.

| **Parameter** | **Bodyweight Trajectory Groups** | | | | **BMI Trajectory Groups** | | | **Pain Trajectory Groups** | | |
| --- | --- | --- | --- | --- | --- | --- | --- | --- | --- | --- |
|  | **Slightly Low-Normal**  **(*n* = 56)** | **Normal**  **(*n* = 16)** | **High-Normal**  **(*n* = 11)** | **High**  **(*n* = 5)** | **Healthy weight (lower)**  **(*n* = 57)** | **Healthy weight (upper)**  **(*n* = 26)** | **Overweight**  **/Obese**  **(*n* = 5)** | **Mild**  **(*n* = 23)** | **Early High**  **(*n* = 50)** | **High**  **(*n* = 15)** |
| **Age (years old)** | | | | | | | | | | |
| 1–5 | 52 (92.9%) | 2 (12.5%) | 0 (0.0%) | 0 (0.0%) | 41 (71.9%) | 13 (50.0%) | 0 (0.0%) | 18 (78.3%) | 30 (60.0%) | 6 (40.0%) |
| 6–15 | 4 (7.1%) | 14 (87.5%) | 11 (100.0%) | 5 (100.0%) | 16 (28.1%) | 13 (50.0%) | 5 (100.0%) | 5 (21.7%) | 20 (40.0%) | 9 (60.0%) |
| **Sex** | | | | | | | | | | |
| Male | 34 (60.7%) | 11 (68.8%) | 7 (63.6%) | 4 (80.0%) | 35 (61.4%) | 17 (65.4%) | 4 (80.0%) | 14 (60.9%) | 32 (64.0%) | 10 (66.7%) |
| Female | 22 (39.3%) | 5 (31.2%) | 4 (36.4%) | 1 (20.0%) | 22 (38.6%) | 9 (34.6%) | 1 (20.0%) | 9 (39.1%) | 18 (36.0%) | 5 (33.3%) |
| **Ethnicity** | | | | | | | | | | |
| Thai | 43 (76.8%) | 12 (75.0%) | 6 (54.6%) | 4 (80.0%) | 39 (68.4%) | 21 (80.8%) | 5 (100.0%) | 19 (82.6%) | 39 (78.0%) | 7 (46.7%) |
| Non-Thai | 13 (23.2%) | 4 (25.0%) | 5 (45.4%) | 1 (20.0%) | 18 (31.6%) | 5 (19.2%) | 0 (0.0%) | 4 (17.4%) | 11 (22.0%) | 8 (53.3%) |
| **Risk Stratification** | | | | | | | | | | |
| Standard | 42 (75.0%) | 9 (56.3%) | 2 (18.2%) | 0 (0.0%) | 36 (63.1%) | 15 (57.7%) | 2 (40.0%) | 16 (69.6%) | 30 (60.0%) | 7 (46.7%) |
| High | 11 (19.6%) | 5 (31.2%) | 6 (54.5%) | 5 (100.0%) | 16 (28.1%) | 8 (30.8%) | 3 (60.0%) | 5 (21.7%) | 17 (34.0%) | 5 (33.3%) |
| Very high | 3 (5.4%) | 2 (12.5%) | 3 (27.3%) | 0 (0.0%) | 5 (8.8%) | 3 (11.5%) | 0 (0.0%) | 2 (8.7%) | 3 (6.0%) | 3 (20.0%) |

**Supplementary Table 2.** Classification for bodyweight z-scores.

| **Class** | **Weight Z-score** |
| --- | --- |
| Low weight | Below -2.0 |
| Slightly low weight | -2.0 to -1.5 |
| Low normal weight | -1.5 to -1.0 |
| Slightly low-normal weight | -1.0 to -0.5 |
| Normal weight | -0.5 to 0.5 |
| Slightly high-normal weight | 0.5 to 1.0 |
| High-normal weight | 1.0 to 1.5 |
| Slightly high weight | 1.5 to 2.0 |
| High weight | Above 2.0 |

**Supplementary Table 3.** Classification for BMI z-scores

| **Class** | **BMI Z-score** |
| --- | --- |
| Underweight | Below -2.0 |
| Slightly thin | -2.0 to -1.0 |
| Healthy weight | -1.0 to 1.0 |
| Slightly overweight | 1.0 to 2.0 |
| Overweight/obese | Above 2.0 |
